# Impact of the drug-coated balloon-to-predilation balloon diameter ratio on restenosis risk in hemodialysis access: a study of nonlinear association

**DOI:** 10.1080/0886022X.2026.2640723

**Published:** 2026-03-09

**Authors:** Aiqiang Zhou, Yong Lu, Liangzhu Hu, Chao Wang, Yafei Zi, Yedong He, Tao Huang, Luxiang Wen, Yangdong Liu

**Affiliations:** a Department of Vascular and Endovascular Surgery, South China Hospital, Medical School, Shenzhen University, Shenzhen, P. R. China; b Department of Vascular and Endovascular Surgery, Zhangping City Hospital, Zhangping, Fujian, P. R. China

**Keywords:** Drug-coated balloon, arteriovenous access, balloon angioplasty, device sizing, hemodialysis

## Abstract

**Background:**

Drug-coated balloons (DCBs) improve the patency of hemodialysis access, yet restenosis rates remain substantial. This may be partly attributable to suboptimal balloon sizing. The relationship between the balloon diameter ratio (BDR; the ratio of DCB diameter to final predilation balloon diameter) and restenosis risk has not been clearly elucidated.

**Methods:**

This retrospective study analyzed 123 patients who underwent DCB angioplasty for dysfunctional arteriovenous fistulas (AVF) or arteriovenous grafts (AVG). The primary endpoint was clinically driven target lesion restenosis within six months. Multivariable Cox regression and restricted cubic spline (RCS) analyses were used to evaluate the association between BDR and restenosis.

**Results:**

The six-month restenosis rate was 25.2%. BDR was an independent predictor of restenosis (*p* = 0.048). Compared to a BDR of 1.0 (a perfect 1:1 match), a BDR < 1 was associated with a significantly higher risk (HR 3.07, 95% CI 1.01–9.34). RCS analysis demonstrated a significant U-shaped nonlinear relationship (*P* for nonlinearity = 0.041), with the risk nadir observed at a BDR of approximately 0.98. A BDR within the 0.9–1.1 range constituted a “safe window” associated with the lowest risk of restenosis.

**Conclusions:**

BDR is a key, modifiable technical factor influencing patency after hemodialysis access intervention. Maintaining a BDR within the 0.9–1.1 range correlates with the lowest 6–month restenosis risk, yielding a clear and practical technical target for optimizing DCB angioplasty procedures.

## Introduction

Hemodialysis (HD) remains the primary treatment for end–stage renal disease. According to KDOQI guidelines, an arteriovenous fistula (AVF) is the preferred form of vascular access [1]. However, the creation of an AVF alters local hemodynamics, and the resulting shear stress can stimulate endothelial proliferation, leading to stenoses and, ultimately, access failure [2]. Reported primary patency rates for AVFs and arteriovenous grafts (AVGs) are 70 and 48% at 1 and 4 years, respectively [3]. Approximately 30% of AVFs and 50% of AVGs require intervention within six months of initiating dialysis to maintain patency [4]. High-pressure balloon angioplasty is the conventional treatment for access stenosis. However, its one-year primary patency rates are modest, ranging from 40 to 60% for AVFs and 20–40% for AVGs [4]. This is largely due to endothelial injury and subsequent neointimal hyperplasia provoked by the balloon dilation [5]. Drug-coated balloons (DCBs), which deliver antiproliferative agents such as paclitaxel, have significantly improved clinical outcomes by attenuating this hyperplastic response [6]. Although recent studies have reported 6-month target lesion patency rates of 85–91% after DCB angioplasty [7,8]. A subset of patients still experiences restenosis within this timeframe. This variability in real-world outcomes may be linked to heterogeneity in procedural technique, particularly balloon sizing. Current local drug delivery strategies for AVF stenosis can be broadly classified into two categories: intraluminal delivery, exemplified by DCBs, and the emerging perivascular wrap-based sustained release strategy, in which drug-eluting polymers are placed around the target vessel to achieve long-term localized therapeutic effects. Although DCBs enable direct drug transfer to the intima, their efficacy can be compromised by inefficient drug penetration into the vessel wall and rapid washout by high blood flow [4,9]. The perivascular approach offers an alternative paradigm. For instance, Klusman et al. [10] integrated rosuvastatin-loaded gold nanoparticles into a polycaprolactone wrap, while Barcena et al. [11–13] reported on wraps eluting different anti-proliferative agents, incorporating gold or bismuth nanoparticles to facilitate longitudinal CT monitoring. These innovative strategies represent promising new avenues for sustained inhibition of intimal hyperplasia. Nevertheless, DCBs remain the most mature and widely utilized technology in contemporary clinical practice. Optimizing their procedural details is thus of immediate clinical relevance for improving patient outcomes. Given the variability in balloon selection and the observation that restenosis can still occur post-DCB, this study focused on a specific, modifiable procedural parameter: balloon sizing. We aimed to investigate the relationship between the DCB-to-predilation balloon diameter ratio (BDR) and the risk of restenosis.

## Methods

### Study design and population

This single-center retrospective study enrolled patients with symptomatic AVF/AVG stenosis who underwent DCB angioplasty at the Department of Vascular and Endovascular Surgery, Shenzhen University General Hospital, from September 1, 2022, to June 30, 2025. Inclusion criteria were: 1. A mature upper or lower extremity AVF/AVG stenosis confirmed by ultrasound that required percutaneous transluminal angioplasty (PTA); 2. Treatment with a DCB. Exclusion criteria were: 1. Intraoperative or prior stent placement; 2. Concomitant open surgery; 3. Fistula infection; 4. Central venous stenosis/occlusion; 5. The use of more than two predilation balloons for a single target lesion. This study was conducted in accordance with the Declaration of Helsinki and approved by the Institutional Ethics Committee of Shenzhen University General Hospital (Approval No. HNLS2025003-A). All patients provided written informed consent.

### Procedures

Preoperative duplex ultrasound was performed to evaluate lesion characteristics, stenosis severity, and reference vessel diameter. Heparin (50 IU/kg) was administered routinely before PTA. All cases underwent predilation with a non-DCB balloon. The balloon type (high-pressure, scoring, or ultra-high-pressure) and diameter were selected by the operator based on lesion morphology and the reference vessel diameter; for severe stenoses, balloon diameter was increased incrementally. Following predilation, intraprocedural angiography was performed to confirm luminal improvement of the target lesion. A DCB was then advanced to the target lesion and inflated for 3 min, followed by a final angiographic assessment. A single dose of low-molecular-weight heparin (4000–6000 U) was given postprocedurally. Clinical and ultrasound evaluations were performed on postoperative day 1. Subsequent follow-up was performed every 3–6 months with duplex ultrasound, venography, or telephone interview, or as clinically indicated.

### Definitions

BDR was defined as the ratio of the nominal diameter of the DCB to that of the largest predilation balloon used for the index procedure. The primary endpoint was target lesion restenosis within 180 days, defined as angiographically confirmed luminal stenosis >50% associated with clinical or functional deterioration (e.g., reduced dialysis adequacy, elevated venous pressure during hemodialysis). Target lesion primary patency was defined as the time from the index procedure to the first clinically driven target lesion reintervention or radiologically confirmed restenosis. Technical success was defined as residual stenosis ≤ 30% on the postprocedural angiogram without flow-limiting dissection. Clinical success was defined as the ability to successfully perform dialysis using the access, accompanied by relief of symptoms related to venous hypertension.

### Data collection and statistical analysis

Data on patient demographics, clinical characteristics, and procedural details were collected (specific variables are listed in Table 1). All statistical analyses were performed using R software. Continuous variables were reported as mean ± standard deviation or median (interquartile range, IQR), and categorical variables as frequencies (percentages). Group comparisons were performed using the *t*-test, Mann-Whitney *U* test, or chi-square test, as appropriate. Survival analysis was conducted using the Kaplan-Meier method with the log-rank test. Multivariable Cox proportional hazards regression was performed to adjust for relevant confounders and calculate hazard ratios (HRs) with 95% confidence intervals (CIs). Restricted cubic spline (RCS) analysis was used to explore the nonlinear relationship between BDR and restenosis, with the reference value set at a BDR of 1.0. A two-tailed *p*-value < 0.05 was considered statistically significant.

**Table 1.** Baseline clinical and surgical characteristics of patients stratified by BDR.

Characteristic	Overall (*n* = 123)	BDR <1 (*n* = 11)	BDR =1 (*n* = 82)	BDR > 1 (*n* = 30)	*p*
Demographics					
Age, years, mean (SD)	52.4 (12.2)	53.5 (13.5)	51.6 (11.7)	54.0 (12.7)	0.628
Male, *n* (%)	68 (55.3)	8 (72.7)	44 (53.7)	16 (53.3)	0.475
Comorbidities, *n* (%)					
Diabetes	44 (35.8)	3 (27.3)	27 (32.9)	14 (46.7)	0.335
Hypertension	70 (56.9)	7 (63.6)	41 (50.0)	22 (73.3)	0.078
Laboratory Values					
Albumin (ALB), g/L, mean (SD)	43.2 (3.8)	43.2 (3.7)	43.2 (4.0)	43.2 (3.3)	0.998
Hemoglobin (HB), g/L, median [IQR]	122.0 [112.0, 134.0]	128.0 [97.0, 141.0]	122.0 [114.0, 134.0]	120.0 [112.0, 131.0]	0.759
LDL, mmol/L, median [IQR]	2.33 [2.05, 2.57]	2.29 [2.02, 2.59]	2.33 [2.03, 2.54]	2.31 [2.16, 2.60]	0.709
Platelet count (PLT), ×10⁹/L, median [IQR]	185.0 [144.0, 240.0]	184.0 [141.0, 229.0]	194.0 [145.0, 244.0]	178.0 [142.0, 240.0]	0.877
Access and lesion characteristics					
AVF/AVG type, *n* (%)					0.153
AVF	111 (90.2)	9 (81.8)	77 (93.9)	25 (83.3)	
AVG	12 (9.8)	2 (18.2)	5 (6.1)	5 (16.7)	
Side, *n* (%)					0.93
Left	95 (77.2)	9 (81.8)	63 (76.8)	23 (76.7)	
Right	28 (22.8)	2 (18.2)	19 (23.2)	7 (23.3)	
Stenosis diameter, mm, median [IQR]	1.70 [1.30, 2.30]	1.50 [1.10, 1.90]	1.70 [1.40, 2.30]	1.60 [1.20, 2.20]	0.313
Normal Vein diameter, mm, median [IQR]	6.60 [5.90, 7.30]	6.70 [5.90, 7.50]	6.60 [5.90, 7.40]	6.60 [6.00, 7.00]	0.861
Stenosis degree, %, mean (SD)	73.2 (9.0)	77.0 (6.1)	72.5 (8.8)	73.5 (9.8)	0.287
Previous PTA times, median [IQR]	1.0 [0.0, 3.0]	2.0 [1.0, 5.0]	1.0 [0.0, 3.0]	1.0 [0.0, 2.0]	0.083
Access sustained time, months, median [IQR]	30.0 [11.0, 58.0]	25.0 [19.0, 60.0]	32.0 [11.0, 54.0]	26.0 [10.0, 48.0]	0.79
Procedural details					
Pre-dilation balloon type, *n* (%)					0.026
Group 1 (High-pressure)	67 (54.5)	7 (63.6)	40 (48.8)	20 (66.7)	
Group 2 (Scoring)	46 (37.4)	1 (9.1)	36 (43.9)	9 (30.0)	
Group 3 (Other)	10 (8.1)	3 (27.3)	6 (7.3)	1 (3.3)	
Pre-dilation Balloon diameter, mm, median [IQR]	6.00 [6.00, 7.00]	7.00 [6.00, 8.00]	6.00 [6.00, 7.00]	5.00 [5.00, 6.00]	<0.001
DCB Type Group, *n* (%)					0.324
Group 1	61 (49.6)	3 (27.3)	41 (50.0)	17 (56.7)	
Group 2	47 (38.2)	5 (45.5)	33 (40.2)	9 (30.0)	
Group 3	15 (12.2)	3 (27.3)	8 (9.8)	4 (13.3)	
DCB diameter, mm, median [IQR]	6.00 [6.00, 7.00]	6.00 [5.00, 6.00]	6.00 [6.00, 7.00]	6.00 [6.00, 7.00]	0.042

Note: Categorical variables are expressed as *n* (%). Continuous variables are presented as mean (standard deviation) or median [interquartile range] based on their distribution characteristics. P-values were calculated using analysis of variance (ANOVA), Kruskal-Wallis test, or chi-square test as appropriate. In the drug-eluting balloon grouping: Group 1: UltraFree^™^ paclitaxel-coated balloon (Journey Medical, China); Group 2: Aperto^®^ paclitaxel-coated balloon (Xianruida, China); Group 3: DCB balloons from other manufacturers.

## Results

A total of 123 consecutive patients who underwent DCB angioplasty were included in the final analysis. Technical and clinical success were both 100%. The mean patient age was 52.4 years, and 55.3% were male. The median lesion diameter was 1.70 mm, the median normal vein diameter was 6.60 mm, and the mean stenosis was 73.2%. The median access age was 30 months. The six-month primary patency rate was 74.8%, representing a restenosis rate of 25.2% (31/123). For the present analysis, a BDR of 1.00 was defined as an exact 1:1 nominal diameter match between the final predilation balloon and the DCB. Patients were categorized accordingly: BDR < 1 (*n* = 11), BDR = 1 (*n* = 82), and BDR > 1 (*n* = 30). Baseline clinical characteristics were well-balanced across the groups **(**Table 1**)**. Regarding procedural details, significant differences were observed in the type of predilation balloon used (*p* = 0.026), with high-pressure balloons (Group 1) being the most common (54.5%). Significant differences were also observed in predilation balloon diameter (*p* < 0.001) and DCB usage across the BDR subgroups.

**Figure 1.**
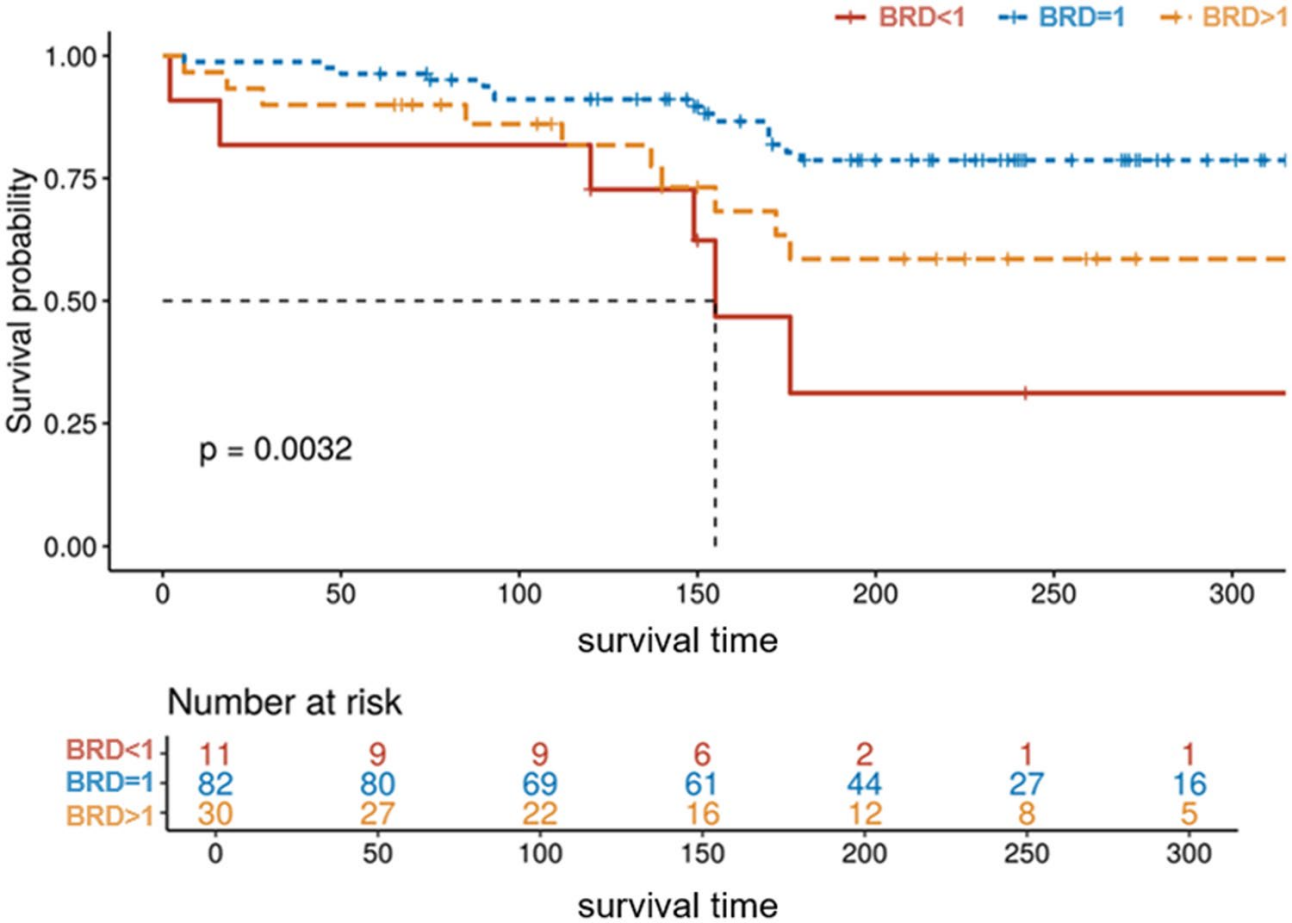
Kaplan-Meier curves for restenosis-free survival stratified by BDR. Patients were divided into three groups: BDR < 1, BDR = 1, and BDR > 1. Survival curves demonstrated significant differences among groups during the 180-day follow-up period (Log-rank test, *p* = 0.0032), with the BDR = 1 subgroup exhibiting the best survival outcomes. The number of patients at risk at each time point is shown in the table below the figure.

As shown in Table 2, BDR was a significant predictor of the 180-day target lesion restenosis rate. The restenosis rates differed significantly among the three groups (*p* = 0.017). The BDR = 1 group demonstrated the best efficacy, with the lowest 180-day restenosis rate of 18.3% (15/82). In contrast, the BDR < 1 group exhibited the highest risk, with a restenosis rate of 54.5% (6/11) and a restenosis-free survival rate of only 45.5%. The BDR > 1 group showed an intermediate risk, with a restenosis rate of 33.3% (10/30) and a restenosis-free survival rate of 66.7%. Kaplan-Meier analysis revealed significant differences in restenosis-free survival among the three groups (log-rank test, *p* = 0.0032; Figure 1), with the BDR = 1 group demonstrating the most favorable patency.

**Table 2.** Comparison of 180-day restenosis outcomes among patients in different BDR subgroups.

BDR group	Total patients	Restenosis events, *n*	180-day Restenosis rate (%)	180-day Restenosis-free survival (%)	*p* value
BDR < 1	11	6	54.50%	45.50%	0.017
BDR = 1	82	15	18.30%	81.70%	
BDR > 1	30	10	33.30%	66.70%	
Overall	123	31	25.20%	74.80%	

Note: The *p*-value for the difference in restenosis rates between groups was obtained from the chi-square test (*χ*² = 8.153). The *p*-value for the log-rank test in the Kaplan–Meier survival analysis between groups was 0.0032.

Results of the univariable and multivariable Cox regression analyses are detailed in Table 3. Univariable analysis showed that BDR category, access type (AVG), and the use of a scoring balloon for predilation were significantly associated with restenosis. In the multivariable model, after adjusting for access type, predilation balloon type, LDL cholesterol, and normal vein diameter, BDR remained an independent predictor of restenosis (overall *p* = 0.048). Specifically, compared to the BDR = 1 group, the BDR < 1 group had a nearly threefold increase in restenosis risk (adjusted HR = 3.07, 95% CI: 1.01–9.34). The BDR > 1 group also demonstrated a notable trend toward increased restenosis risk, which did not reach statistical significance in the categorical analysis (adjusted HR = 1.76, 95% CI: 0.77–4.03).

**Table 3.** Univariate and multivariate cox proportional hazards regression an alysis for restenosis.

	Univariate analysis		Multivariable analysis	
Variable	HR (95% CI)	*P* value	Adjusted HR (95% CI)	*P* value
BDR		**<0.01**		**0.048**
BDR < 1	4.29 (1.65–11.14)	**0.003**	3.07 (1.01–9.34)	**0.048**
BDR =1 (Reference)	1	**–**	1	–
BDR > 1	2.30 (1.03–5.12)	**0.042**	1.76 (0.77–4.03)	0.18
Age	1.00 (0.97–1.03)	0.91		
Sex (Male)	1.05 (0.52–2.13)	0.89		
Diabetes	0.87 (0.41–1.84)	0.711		
Hypertension	1.36 (0.65–2.83)	0.416		
Albumin (g/L)	1.00 (0.92–1.10)	0.941		
Hemoglobin (g/L)	0.99 (0.97–1.01)	0.411		
LDL-C (mmol/L)	0.66 (0.34–1.29)	0.229	0.76 (0.37–1.56)	0.456
Platelet count (×10⁹/L)	1.00 (1.00–1.01)	0.847		
Access type (AVG)	5.32 (2.43–11.63)	**<0.001**	6.33 (2.49–16.13)	**<0.001**
Side (Right)	1.04 (0.45–2.41)	0.935		
Stenosis diameter (mm)	0.92 (0.60–1.42)	0.699		
Stenosis degree (%)	0.34 (0.01–18.71)	0.599		
Normal Vein diameter (mm)	0.83 (0.65–1.08)	0.166	0.72 (0.53–0.97)	**0.031**
Previous PTA times	1.10 (0.95–1.27)	0.205		
Access maturation time (months)	1.00 (0.99–1.01)	0.533		
Pre-dilation balloon type		**0.028**		0.089
High-pressure (Reference)	1	–		
Scoring balloon	0.30 (0.11–0.78)	**0.014**	0.38 (0.14–1.03)	0.057
Ultra-high-pressure balloon	1.35 (0.46–3.94)	0.58	0.48 (0.12–1.94)	0.300
Pre-dilation balloon diameter (mm)	1.34 (0.83–2.18)	0.233		
DCB balloon diameter (mm)	1.53 (0.87–2.69)	0.144		
DCB type group		0.321		
Ultrafree (Reference)	1	–		
Aperto	0.65 (0.28–1.51)	0.319		
XRDA	1.60 (0.63–4.08)	0.321		

**Figure 2.**
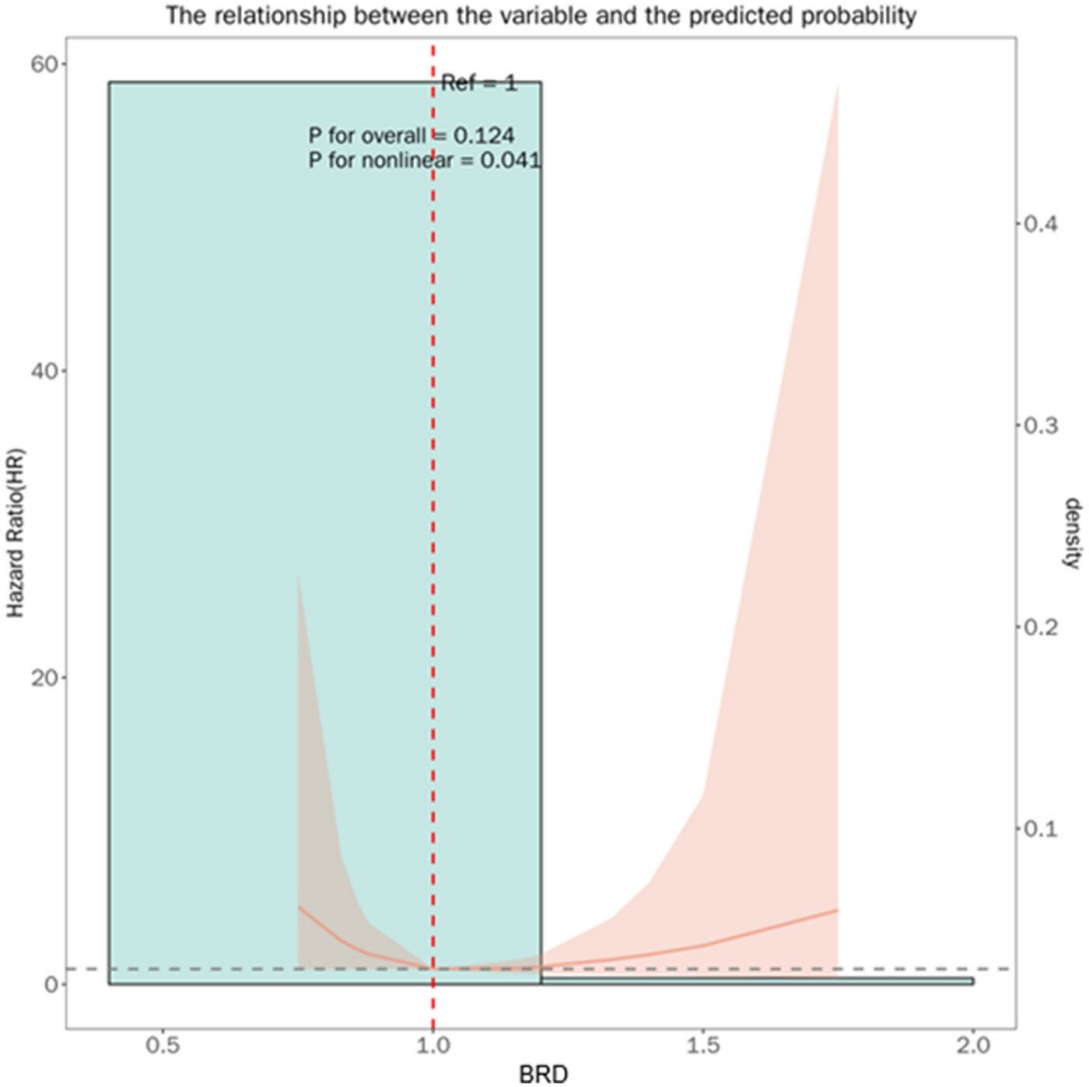
Restricted cubic spline analysis demonstrates the relationship between balloon diameter ratio and the risk of adjusted restenosis within 180 days post-procedure. Solid lines represent hazard ratios, with shaded areas indicating 95% confidence intervals. The reference value is set at BDR = 1.0. The nonlinear *p*-value was 0.041, indicating a significant *U*-shaped association. The risk curve reached its lowest point at a BDR of approximately 0.98. Risk remained low within the BDR range of 0.9 to 1.1, but increased significantly outside this range.

Restricted cubic spline analysis was performed to delineate the precise relationship between BDR and restenosis risk (Figure 2). This analysis revealed a significant nonlinear “U-shaped” relationship (P for nonlinearity = 0.041). The nadir of the risk curve, indicating the lowest restenosis risk, occurred at a BDR of approximately 0.98. The analysis further defined a “safe window” between 0.9 and 1.1, where the risk of restenosis remained low. Deviations from this window in either direction were associated with a significantly increased restenosis risk.

## Discussion

The present study demonstrates that BDR is a critical, modifiable procedural factor associated with restenosis following DCB angioplasty for hemodialysis vascular access. We identified a significant U-shaped, nonlinear relationship between BDR and the six-month risk of restenosis. The risk was lowest when the BDR was maintained within a narrow window of 0.9–1.1, with the absolute lowest point at a perfect 1:1 match (BDR ≈ 1.0). These findings provide a clear, quantifiable target for optimizing DCB procedures. In addition, our analysis confirmed that AVGs are associated with a significantly higher risk of restenosis, while a larger baseline vein diameter is a strong protective factor.

While DCBs have revolutionized the management of access stenosis [14], outcome heterogeneity remains a significant clinical challenge. The 6-month restenosis rate of 25.2% in our study cohort, although favorable compared with historical controls, highlights this issue, particularly given reports of substantially higher failure rates in selected high-risk patient populations [15]. Our study suggests that standardizing technical details, such as optimal balloon sizing, is key to improving overall outcomes. Although the importance of adequate predilation and optimal balloon apposition is well established in the coronary artery literature [16], hemodialysis vascular access poses unique physiological challenges. The high-flow state of an AVF or AVG can drive aggressive intimal hyperplasia [17], and morphological features like intimal thickening (≥ 1.2 mm) have been shown to impede paclitaxel penetration, reducing DCB efficacy [18,19]. These unique characteristics preclude the direct extrapolation of coronary experience to the dialysis population and instead necessitate access-specific optimization of procedural parameters, as demonstrated by the BDR examined in this study [7].

Our findings provide quantitative guidance for the principle of “adequate predilation”. A BDR ≈ 1.0 signifies optimal alignment between the DCB and the prepared lumen. When the BDR is < 1, the balloon may not achieve full, uniform wall contact, leading to heterogeneous drug transfer and insufficient dosage. Conversely, a BDR > 1 represents overdilation. Our data suggest that when BDR exceeds 1.1, the mechanical injury from overdilation may incite a proliferative response that negates the benefit of a larger acute luminal gain. This is consistent with the well-established principle that excessive balloon oversizing may increase the risk of vascular injury and endothelial denudation, which paradoxically predisposes to restenosis [7]. BDR is therefore a key parameter that balances the competing goals of achieving acute luminal gain and minimizing iatrogenic injury. Beyond this macroscopic mechanical balance, BDR likely influences outcomes through more nuanced pharmacokinetic mechanisms. The findings of this study suggest that optimizing local drug pharmacokinetics may be a key pathway through which BDR influences outcomes. When a DCB is well-approxiamted to the vessel wall (BDR ≈ 1), the transfer of paclitaxel to the tissue is likely more homogeneous and efficient. Furthermore, optimal apposition may protect the drug coating from immediate washout by the high-shear hemodialysis access flow, improving drug retention [9]. In contrast, incomplete apposition (BDR < 1) can lead to heterogeneous drug distribution and underdosing of the target lesion, while overdilation (BDR > 1) may cause sufficient endothelial denudation to paradoxically hinder effective drug uptake and retention.

Our multivariable analysis also sheds light on the profound impact of baseline patient and access characteristics. The strong association between AVG and restenosis is multifactorial. First, the expanded polytetrafluoroethylene (ePTFE) material itself acts as a permanent foreign body, inciting a chronic inflammatory response that drives smooth muscle cell and fibroblast proliferation [20]; second, the compliance mismatch between the graft and native vessel creates turbulent flow, low wall shear stress, and high circumferential stress at the anastomosis, all of which are potent mitogenic signals [21]. Third, these processes ultimately promote a synthetic phenotype shift in smooth muscle cells, leading to stenotic lesions rich in extracellular matrix [21]. As DCBs primarily inhibit cellular proliferation [22], their efficacy may be blunted against these matrix-rich, AVG-specific lesions. Conversely, a larger native vein diameter is protective. A larger caliber vessel maintains higher, more physiologic shear stress, which upregulates anti-proliferative pathways like TGF-β1. Additionally, a larger native vein confers greater injury tolerance, as a given balloon diameter results in less relative overstretching and mural vascular injury, thereby eliciting a more blunted healing response [21,23].

Several other procedural variables may modulate DCB efficacy, including balloon inflation duration; device-specific characteristics like coating technology and excipient type; and pharmacotherapy such as antiplatelet agents [4,9,24]. These factors were not standardized in our retrospective cohort; however, based on the present findings, we propose the following actionable procedural recommendations: 1. Precise Preprocedural Planning: Perform duplex ultrasound to accurately measure the diameter of the normal native vessel adjacent to the target lesion to guide individual balloon sizing. 2. Adequate Predilation: The primary goal of predilation is to create a stable, fully dilated luminal space to facilitate subsequent DCB delivery and optimal apposition to the vessel wall. 3. Adhere to the 1:1 Matching Principle: Select a DCB with a nominal diameter as close as possible to that of the final predilation balloon for the target lesion. Target a BDR within the 0.9–1.1 safe window, with a BDR of 1.0 as the ideal procedural target. 4. Identify High-Risk Patients: Patients with AVGs or small-caliber native veins should be identified as being at high risk of restenosis and managed with a more intensive post-procedural surveillance protocol.

Although our study includes a diverse range of device types, which reflects real-world clinical practice, it has several important limitations that warrant acknowledgment. The first is the retrospective, single-center design, which introduces potential for selection bias and unmeasured confounding from lesion-specific factors (e.g., calcification, length, location, *de novo* vs. recurrent status, and intimal thickness) and operator experience. A second major limitation is the small size of the BDR < 1 subgroup (*n* = 11). This results in a wide confidence interval for the hazard ratio, and while statistically significant, this finding is statistically fragile and requires validation in larger cohorts. Third, due to limited sample sizes, we could not perform subgroup analyses by device type or access type (AVF vs. AVG), despite their known pathophysiological differences. Future adequately powered, prospective, multicenter studies are needed to validate our findings, to systematically assess the impact of lesion morphology, and to determine if the optimal BDR window differs between AVFs and AVGs or across various device platforms.

## Conclusion

The present study demonstrates that BDR is a key modifiable procedural factor associated with vascular patency following endovascular intervention for hemodialysis arteriovenous access. A clear U-shaped relationship exists between BDR and restenosis risk, with optimal outcomes achieved when the ratio is maintained within the 0.9–1.1 range. Additionally, AVG and smaller native vein diameters are strong predictors of restenosis. Therefore, to maximize DCB efficacy, precise intraoperative BDR matching is recommended as a standard procedural practice, in conjunction with enhanced postprocedural follow-up for patients with AVGs or poor baseline vascular characteristics.

## Data Availability

The datasets generated during and/or analyzed during the current study are not publicly available due to patient privacy and ethical restrictions but are available from the corresponding author on reasonable request.
